# Perspectives on managing skin manifestations in cancer patients: a multidisciplinary mixed-method survey of oncologists and dermatologists

**DOI:** 10.1007/s00520-025-10254-w

**Published:** 2026-01-11

**Authors:** Davide Fattore, Omalkhair Abulkhair, Ola M. Reda Khorshid, Tanios Bou Khalil, Rania H. Kafrouny, Michele Cherfane

**Affiliations:** 1 Section of Dermatology, Department of Clinical Medicine and Surgery, University of Naples Federico II, Naples, Italy; 2 Medical Oncology Department, Oncology Services, Alhabib Hospital, Riyad, Saudi Arabia; 3 Department of Medical Oncology, National Cancer Institute, NCI, Cairo University, Cairo, Egypt; 4 Pierre Fabre Laboratories, Middle East, Beirut, Lebanon; 5 INSPECT-LB (Institut National de Santé Publique, Épidémiologie Clinique et de Toxicologie-Liban), Beirut, Lebanon; 6 Gilbert and Rose-Marie Chagoury School of Medicine, Lebanese American University, Byblos, Lebanon

**Keywords:** Oncology, Onco-dermatology, Adverse events, Skin manifestations, Quality of life

## Abstract

**Background:**

Cancer treatments often cause skin toxicities that disrupt therapy and reduce patients’ quality of life, yet research on their management in the Middle East is scarce. This study aims to examine the practices and challenges of managing skin-related adverse effects in cancer patients.

**Methods:**

A mixed-method survey was conducted on a sample of dermatologists and oncologists practicing in Middle Eastern countries who were invited to participate in Pierre Fabre's annual Middle East Medical Convention. A brief online questionnaire, distributed to 250 dermatologists and 19 oncologists, using both quantitative and qualitative approaches, assessed the frequency, types, and management of skin conditions, alongside perceived barriers and referral practices.

**Results:**

The study included responses from 117 dermatologists and 19 oncologists. Oncologists encountered cancer-related skin manifestations considerably more often, with 44.4% seeing such cases daily compared to 8.1% of dermatologists (*p* < 0.001). Notable differences were also observed in the types of skin conditions reported, such as nail changes, which were significantly more frequent among dermatologists (72.6% vs. 31.6%, *p* < 0.001), whereas hand-foot syndrome was markedly more commonly observed among oncologists (89.5% vs. 6.0%, *p* < 0.001). Dermatologists reported feeling very comfortable managing these conditions more often than oncologists (50.4% vs. 10.5%, *p* = 0.004). Key barriers included the severity of skin conditions, selecting appropriate dermo-cosmetic products, and limited knowledge in onco-dermatology. The qualitative analysis highlighted oncologists’ challenges in managing skin toxicities under three main themes: (1) preventing cancer treatment interruptions, (2) limited specialized dermatologic knowledge and support, and (3) addressing patient-centered issues.

**Conclusion:**

Findings reveal differing perspectives between dermatologists and oncologists and underscore the need for greater collaboration to optimize the management of cancer-related skin toxicities.

**Supplementary Information:**

The online version contains supplementary material available at 10.1007/s00520-025-10254-w.

## Background

Cancer patients often face a complex array of health challenges, including dermatologic disorders that arise as side effects of oncological treatments such as chemotherapy, radiation, immunotherapy, and targeted therapies [1–3]. These dermatologic complications manifest as a wide range of conditions, including dry skin, flushing, hair loss, hyperpigmentation, nail changes, photosensitivity, pruritus, papulopustular eruption, hand-foot syndrome, and mucositis [4, 5]. Managing dermatologic complications in cancer patients poses several challenges. First, skin toxicities are highly prevalent in oncology patients during anticancer treatments, with severity varying from mild irritations to severe reactions and often associated with a significant physical burden [6]. Second, these conditions may be regarded as minor complaints compared to cancer symptoms, but they can severely impair emotional and interpersonal well-being and negatively affect the quality of life [7–9]. Third, skin toxicities may result in treatment interruptions or dose modifications, which can adversely influence the overall effectiveness of cancer treatment and patient outcomes [10].

The management of these manifestations requires specialized knowledge of both oncology and dermatology. However, there is a recognized gap in dermatologic care practices among oncologists, who may lack training in managing skin-related toxicities [11, 12]. Similarly, while dermatologists play a vital role in managing skin conditions, they are often unaware of cancer therapies and may benefit from additional expertise to address the specific challenges associated with cancer-related skin manifestations and the effects of anti-tumor therapies [13, 14]. In response to these challenges, the field of onco-dermatology has emerged to bridge the gap between oncology and dermatology, fostering collaboration between the two specialties [15]. Onco-dermatology emphasizes the integration of dermatologic care into oncology practices to improve the management of skin toxicities and enhance patient care [16, 17]. This interdisciplinary approach aims to address the specific needs of cancer patients with skin complications, contributing to more comprehensive cancer treatment strategies [17, 18].

International guidelines, such as those from the American Society of Clinical Oncology (ASCO) [19] and the European Society for Medical Oncology (ESMO) [12], aim to bridge the gap in dermatologic care within oncology by providing recommendations for managing dermatologic toxicities in cancer patients. Another international consensus, developed jointly by the Association Francophone des Soins Oncologiques de Support (AFSOS) and the Multinational Association of Supportive Care in Cancer (MASCC), provides recommendations on the use of dermo-cosmetic products [14]. In addition, many publications offer guidance on specific drug-induced skin toxicities, such as the European Academy of Dermatology and Venereology (EADV) dermatology for cancer patients’ task force’s position statement, which outlines recommendations for managing dermatologic adverse events caused by immune checkpoint inhibitors [20]. Furthermore, regional guidelines, such as those from Australia and New Zealand [4], also contribute to the global efforts to standardize care.

However, recommendations or standardized protocols for managing skin manifestations in oncology patients in the Middle East remain scarce. In addition, onco-dermatology remains an emerging and largely undeveloped subspecialty in the Middle East, with no formal training programs or dedicated onco-dermatologists available in most countries of the region. Consequently, cancer patients with dermatologic toxicities are typically managed either by their treating oncologists or by general dermatologists through referral. This context highlights the importance of understanding how both groups currently approach the management of skin manifestations in cancer patients. Furthermore, the Middle East faces distinct systemic and structural challenges, including limited access to specialized onco-dermatology services, the absence of region-specific guidelines, variability in healthcare infrastructure, and inconsistent availability or reimbursement of dermo-cosmetic products. These contextual factors may influence how dermatologists and oncologists manage cancer-related skin toxicities.

To our knowledge, no previous studies have examined how dermatologists and oncologists in the Middle East manage cancer-related skin toxicities or the barriers they face in providing optimal care. Thus, the objective of this study is to evaluate the current practices and challenges faced by dermatologists and oncologists in the management of skin manifestations in cancer patients in the Middle East. This research is an essential first step toward enhancing collaboration between these two specialties in the region and developing initiatives to improve knowledge and practice, while identifying practice gaps and guiding the establishment of regional onco-dermatology initiatives.

## Methods

### Study design and setting

This study employed a mixed-methods design, incorporating both quantitative and qualitative approaches to explore the perspectives of dermatologists and oncologists on managing skin manifestations in cancer patients. The study was conducted in April 2024, prior to the 4th edition of Pierre Fabre Laboratories' annual Middle East Medical Convention (MEMC) on “New Ways to Care”, a continuing medical education meeting primarily targeting clinical dermatologists, focusing on advances in dermatology, with oncologists invited to attend a dedicated session on skin and cancer.

### Study participants and sampling

Participants were recruited using convenience sampling from the list of specialists who had agreed to take part in the MEMC 2024. The invitation to participate in the survey was sent and received by all 250 dermatologists and 19 oncologists who had confirmed their attendance at the event. Dermatologists were drawn from various Middle Eastern countries, including Saudi Arabia, the United Arab Emirates, Lebanon, Kuwait, Qatar, Bahrain, Jordan, Oman, and Iraq, while oncologists were from Syria, Lebanon, Saudi Arabia, and Egypt. The doctors represented diverse areas of practice, including both academic and non-academic clinical dermatologists with broad clinical practice.

### Procedure

Data were collected using a brief online questionnaire designed to gather both quantitative and qualitative information, and is available in the supplementary material. The questionnaire took approximately 5 minutes to complete, and implied consent was obtained by the voluntary completion of the survey.

The quantitative component included 5 multiple-choice questions designed to collect data on 1) the frequency of encountering cancer patients with skin manifestations, 2) the frequency of observing the most common chemotherapy-related skin conditions (dry skin, nail changes, photosensitivity, skin discoloration, hair loss, scars, and hand-foot syndrome), 3) the comfort level, 4) the referral practices, and 5) the barriers faced in managing these skin conditions, including concerns about the severity of the condition, inappropriate treatment choices, lack of oncology training, and limited knowledge of cancer-friendly dermo-cosmetic products. Oncologists were also asked to assess how frequently they discontinued anti-cancer treatments due to severe skin manifestations. In addition, they were asked to qualitatively list the top three challenges they face in managing skin manifestations in their cancer patients. This open-ended question allowed oncologists to provide detailed insights into the complexities of treating these patients.

### Data analysis

The collected data were available as de-identified electronic data in Microsoft Excel spreadsheets. The quantitative data collected were analyzed using SPSS version 25.0. Descriptive statistics, including frequency distributions and counts, were used to summarize the responses of dermatologists and oncologists. Bivariate analysis was performed using chi-square tests or Fisher’s exact tests where appropriate, with a significance level set at *p* < 0.05.

The qualitative data were analyzed using principles of Grounded Theory [21] to identify key challenges in managing skin manifestations in cancer patients. Thematic content analysis [22] was performed in three phases: pre-analysis, exploratory analysis, and data analysis. The responses were first organized for a preliminary reading, followed by careful review of each transcript to establish codes and build themes. Emerging themes were categorized and interpreted, with supporting quotes captured to illustrate each theme. Two members of the research team coded the transcripts, adding additional codes iteratively until data saturation was reached. Differences in coding were resolved through team discussions until consensus was achieved. The qualitative findings were organized and presented using Microsoft Excel.

## Results

A total of 117 dermatologists (response rate 46.8%) and 19 oncologists (response rate 100%) participated in the study. A comparative overview of the patterns of practice in managing skin manifestations among dermatologists and oncologists is presented in Table 1. A statistically significant difference (*p* < 0.001) exists in the frequency of skin manifestations encountered by the two groups. Dermatologists reported infrequent encounters, with 34.2% noting they "rarely" see skin manifestations in cancer patients and 25.6% encountering them "every quarter." In contrast, oncologists observed these manifestations more frequently, with 44.4% encountering skin issues daily, 11.1% weekly, and 30% monthly.

**Table 1:** Practice patterns in managing skin manifestations among dermatologists and oncologists

	All	Dermatologists	Oncologists	*P* value
	136 (100)	117 (86.0)	19 (14.0)	
Frequency of skin manifestation				< 0.001*
*Rarely*	40 (29.6)	40 (34.2)	0 (0)	
*Every Quarter*	32 (23.7)	30 (25.6)	2 (11.1)	
*Monthly*	39 (28.9)	33 (28.2)	6 (33.3)	
*Weekly*	13 (9.6)	11 (9.4)	2 (11.1)	
*Daily*	11 (8.1)	3 (2.6)	8 (44.4)	
Level of comfort in managing skin manifestations				0.004
*Not at all*	7 (5.1)	6 (5.1)	1 (5.3)	
*Somehow/ somewhat comfortable*	68 (50.0)	52 (44.4)	16 (84.2)	
*Very comfortable*	61 (44.9)	59 (50.4)	2 (10.5)	
Barriers to treat				0.303
*Severity of the condition*	59 (49.6)	51 (50.5)	8 (44.4)	
*Inappropriate choice of dermo-cosmetics products*	36 (30.6)	28 (27.7)	8 (44.4)	
*Lack of knowledge in onco-dermatology*	24 (20.2)	22 (21.8)	2 (11.1)	

*Fisher’s exact test used, chi-square test for the rest

Results reported in frequency and proportion *N* (%)

The level of comfort in managing skin side effects of anticancer treatments varied significantly by specialty (*p* = 0.004). Half of dermatologists (50.4%) reported feeling very comfortable managing these conditions, while 44.4% considered themselves "somehow/ somewhat comfortable." In contrast, only 10.5% of oncologists felt very comfortable, with the majority (84.2%) identifying as "somehow/ somewhat comfortable."

**Fig. 1:**
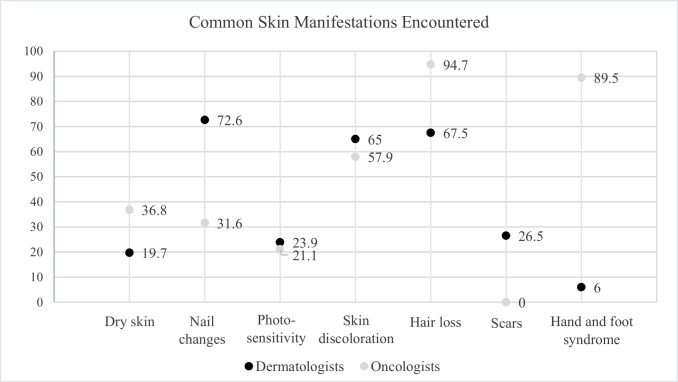
Common skin manifestations encountered by dermatologists and oncologists. *p* = 0.132 for dry skin; *p* < 0.001 for nail changes; *p* = 0.784* for photosensitivity; *p* = 0.552 for skin discoloration; *p* = 0.015 for hair loss; *p* = 0.007* for scars; *p* < 0.001* for hand and foot syndrome, *Fisher exact test used, chi-square test for the rest. Results reported in %

In terms of specific types of skin manifestations, notable differences were found as shown in Fig. 1. Oncologists reported encountering dry skin more frequently (36.8%) compared to dermatologists (19.7%) (*p* = 0.132). Conversely, dermatologists encountered skin discoloration more often (65.0%) than oncologists (57.9%) (*p* = 0.552). Although some numerical differences were observed, they were not statistically significant. In contrast, nail changes were statistically significantly more commonly encountered among dermatologists (72.6%) than oncologists (31.6%) (*p* < 0.001), while scars were reported only by dermatologists (26.5%) (*p* = 0.007). Hair loss was more frequently reported by oncologists, with 94.7% encountering it compared to 67.5% of dermatologists (*p* = 0.015). Hand and foot syndrome was also more frequently observed in oncologists (89.5%) than dermatologists (6.0%) (*p* < 0.001).

**Fig. 2:**
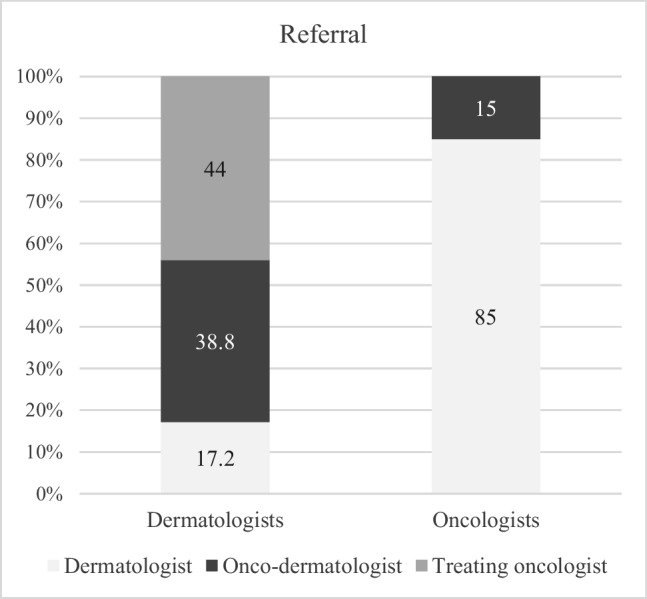
Referral practices for cancer patients with skin manifestations. *P* < 0.0001 between groups

Referral practices differed significantly between the two groups (Fig. 2, *p* < 0.001). Oncologists primarily referred patients to dermatologists (84.2%), while dermatologists exhibited more varied referral patterns, directing patients to onco-dermatologists (38.8%), treating oncologists (44.0%), and other dermatologists (26.7%). None of the participants reported referring patients with skin manifestations to nurses, pharmacists, or general practitioners (GPs).

Regarding treatment barriers, oncologists faced more challenges in selecting appropriate dermo-cosmetic products (44.4%) compared to dermatologists (27.7%), although this difference was not statistically significant (*p* = 0.303). The severity of the skin condition was the most significant barrier reported by both groups, noted by 50.5% of dermatologists and 44.4% of oncologists. Additionally, 21.8% of dermatologists and 11.1% of oncologists cited a lack of knowledge in onco-dermatology as a barrier to effective treatment for cancer patients.

Referral practices based on practitioners’ comfort levels are presented in Table 2. Those who were "not at all" or "somehow/ somewhat comfortable" exhibited similar referral patterns, with no significant preference among dermatologists (32.0%), onco-dermatologists (36.0%), and treating oncologists (32.0%). Conversely, practitioners who felt "very comfortable" showed a higher tendency to refer to treating oncologists (45.0%) or specialized onco-dermatologists (35.0%), while general dermatologists received fewer referrals (20.0%). However, these differences were not statistically significant (*p* = 0.192). Both groups recognized the severity of skin conditions as a primary barrier to treatment, with 58.7% of the very comfortable group and 43.8% of the less comfortable group citing it.

**Table 2:** Referral practices and treatment barriers based on comfort levels in managing skin manifestations

	Not/ somehow/ somewhat comfortable	Very comfortable	*P* value
	76 (55.6)	61 (44.4)	
Specialty of referral to			0.192
*Dermatologist*	24 (32.0)	12 (20.0)	
*Onco-dermatologist*	27 (36.0)	21 (35.0)	
*Treating oncologist*	24 (32.0)	27 (45.0)	
Barriers to treat			0.273
*Severity of the condition*	32 (43.8)	27 (58.7)	
*Inappropriate choice of dermo-cosmetics products*	24 (32.9)	12 (26.1)	
*Lack of knowledge in onco-dermatology*	17 (23.3)	7 (15.2)	

Chi-square test used, Results reported in frequency and proportion *N* (%)

**Fig. 3:**
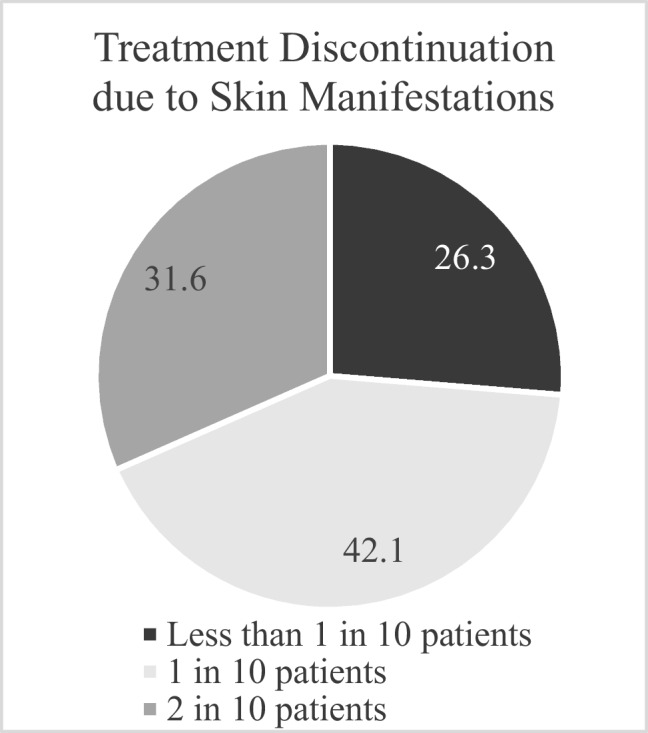
Frequency of cancer treatment discontinuation due to skin manifestations, reported by oncologists

The frequency of cancer treatment discontinuation due to skin manifestations, as reported by oncologists, is illustrated in Fig. 3. Most oncologists (42.1%) indicated that treatment was discontinued due to skin-related issues in approximately 1 in 10 patients.

**Fig. 4:**
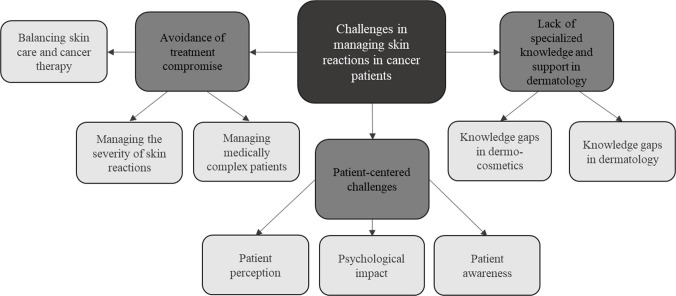
Oncologists’ perceived challenges in managing skin manifestations in cancer patients

The qualitative analysis of the challenges oncologists face in managing skin toxicities identified three key themes (Fig. 4): 1) Avoidance of treatment compromise, 2) Lack of specialized knowledge and support in dermatology, and 3) Patient-centered challenges (Fig. 4). Table 3 provides an overview of these challenges, capturing oncologists’ specific concerns and experiences. In summary, oncologists strive to prevent treatment interruptions due to skin toxicities, expressing concerns about managing the severity of side effects and finding effective treatments. This challenge is exacerbated for medically complex patients with co-existing autoimmune diseases. Balancing skin care with cancer therapy is difficult, with oncologists facing challenges in avoiding severe skin reactions that may necessitate dose reductions. Knowledge gaps in dermatology, particularly regarding the selection of appropriate dermo-cosmetic products, were also highlighted. The absence of onco-dermatologists on-site complicates effective management, as oncologists may have to refer patients outside their institution. Oncologists also identified patient-related challenges, noting that some patients do not consider their skin issues significant enough to mention during the visit, focusing their attention on cancer in that moment, which complicates proactive management. Skin toxicities significantly impact patients’ quality of life, with hair loss being particularly distressing. Some patients perceive dermo-cosmetic treatments as merely esthetic, and the high cost of dermatological products, coupled with the lack of reimbursement, adds to their financial burden.

**Table 3:** Oncologists’ expressed concerns in each key theme challenge

Key themes	Sub-theme	Oncologists’ perceived challenges
Avoidance of treatment compromise due to skin reactions:	Severity of skin reactions and resistance to treatment	“Managing the severity of side effects and finding effective treatments for them is a constant concern” “Some patients do not respond to first-line therapies like hydration and steroids, making skin reactions harder to manage” “Resistance to skin treatments poses a significant obstacle while continuing chemotherapy” “Severe and unpredictable skin complications, are difficult to manage” “Skin toxicities often take a long time to heal, further complicating their management”
	Managing skin reactions in medically complex patients	“Highly compromised patients often experience severe skin diseases, which are difficult to manage” “Co-existing autoimmune diseases complicate the management of skin reactions during treatment”
	Balancing skin care and cancer therapy	“I encounter cases of severe desquamation related to radiation, which complicates radiation treatment” “I face challenges in avoiding severe skin reactions and subsequent dose reduction, or treatment stoppage in my patients” “Some patients refuse to continue their second cycle of treatment because of the skin issues they face” “It’s challenging to minimize interference between skin treatments and anticancer drugs to avoid negatively impacting the overall therapy” “Balancing between skin care and the anti-cancer drug is difficult.”
Lack of specialized knowledge and support in dermatology:	Knowledge gaps in dermo-cosmetics and inappropriate treatments	“I sometimes lack sufficient knowledge about the available skin products on the market” “I worry about administering inappropriate treatments due to this lack of information” “I find it difficult to stay updated on the most recent dermatology therapies, which sometimes results in under-treating skin conditions in my patients.”
	Knowledge gaps in dermatology and lack of on-site expertise	“My institution does not have an onco-dermatologist, and I often have to refer my patients to a dermatologist outside the institute” “Identifying exact skin manifestations (such as allergies, vasculitis, or urticaria) is another challenge I face”
Patient-centered challenges	Patient awareness and preventive practices	“Some patients do not consider their skin issues important enough to mention to me” “I believe that prophylaxis is better than treatment, and I strive to ensure patients use topical creams before radiotherapy to prevent skin toxicities”
	Psychological impact	“I notice that skin complications significantly affect patients' quality of life, often leading to severe psychological distress” “The stigma of hair loss is a particularly distressing issue for patients, affecting their emotional well-being”
	Patient perception and financial burden	“The high cost of dermatological products and lack of reimbursement for these treatments create an additional financial burden for patients” “Some patients perceive skin treatments as purely "esthetic products" and do not prioritize them, which makes it challenging to address and manage skin manifestations effectively during cancer treatment”

## Discussion

Our study highlights notable differences in the frequency, type, and management of skin manifestations encountered by dermatologists and oncologists, while the qualitative analysis further explores the specific challenges oncologists face in managing these skin toxicities in cancer patients.

The findings demonstrate that oncologists more frequently encounter skin-related issues in cancer patients, with a considerable portion observing skin manifestations daily. In contrast, dermatologists report less frequent encounters, likely due to the specialized nature of their practice and fewer direct interactions with cancer patients. Similar findings have been reported in the literature, where oncologists frequently encounter cutaneous toxicities as a consequence of cancer treatments such as chemotherapy and targeted therapies, while dermatologists are often consulted for specific, severe skin conditions in these patients [23–25].

When comparing specific skin conditions, oncologists reported significantly higher encounters of hand and foot syndrome, while dermatologists more frequently observed nail changes and scars. The hand-foot syndrome is a common adverse effect of chemotherapeutic agents, particularly associated with drugs like capecitabine and sorafenib, which are known to cause painful erythema and desquamation in patients undergoing chemotherapy [26, 27]. Oncologists’ higher encounter rates for this condition compared to dermatologists may reflect their daily management of patients receiving these drugs. Dermatologists’ higher identification of nail changes could be due to their expertise in recognizing subtler and more chronic manifestations of skin toxicity not as readily identified by oncologists [24, 28]. Alternatively, it may be patients who do not bring issues to the oncologist’s attention that are instead seen by dermatologists for expertise.

Concerning the reported level of comfort in managing these dermatologic conditions, dermatologists expressed significantly higher comfort levels compared to oncologists, who predominantly referred patients to dermatologists or onco-dermatologists. This finding is consistent with previous studies that emphasize the knowledge gap oncologists experience when managing skin toxicities in cancer patients [11, 12]. Dermatologists, on the other hand, tend to have a higher confidence level due to their specialized training in skin diseases. However, it is noteworthy that only 50% of dermatologists reported feeling "very comfortable" managing skin manifestations in cancer patients, despite their expertise in dermatology. This suggests that while dermatologists are adept at managing general dermatologic conditions, they may not always feel fully equipped to handle the unique challenges posed by cancer patients. This raises an important point that dermatologists would also benefit from further education and training focused specifically on oncology patients, where the interaction between cancer therapies and skin toxicities presents distinct complexities [18, 25, 29].

In terms of treatment barriers, both specialties identified the severity of the skin condition as the primary obstacle. However, oncologists reported more difficulty in selecting appropriate dermo-cosmetic products for cancer patients, a barrier that has been similarly recognized in other studies [30]. While dermo-cosmetics have a recognized role in minimizing skin toxicities and in preventive strategies, these findings should be interpreted within the regional context, as the challenges faced by clinicians in the Middle East differ from those in higher-income settings such as Europe or North America, where specialized onco-dermatology units, standardized care pathways, and broader access to supportive skin care are more established [16–18]. In that context, oncologists’ limited knowledge of dermo-cosmetic options, coupled with the complexity of managing side effects in patients undergoing cancer treatment, underscores the need for clearer guidelines and collaborative care between dermatologists and oncologists. Developing onco-dermatology guidelines could improve both the efficacy of skin toxicity management and patient quality of life [14, 20].

With regards to the challenges expressed by oncologists, first we found that oncologists often struggle to balance effective cancer therapy with the management of dermatologic toxicities. These findings are consistent with existing literature, which emphasizes the challenge of continuing chemotherapy or radiation therapy in the presence of dermatologic side effects. Studies have shown that dermatologic toxicities, such as hand-foot syndrome and severe rash, are significant reasons for dose adjustments or treatment discontinuations in cancer patients [31, 32]. The decision whether or not to suspend antitumor therapy due to skin toxicity requires a broad knowledge of dermatological diseases, their evolution, and their therapy. This is further difficult in the presence of complex medical conditions, highlighting the need for interdisciplinary approaches to manage both the cancer and the associated skin conditions without adversely affecting either. Second, oncologists face an important knowledge gap regarding dermatologic care, particularly in selecting appropriate dermo-cosmetic products for cancer patients. Prior research has similarly identified the need for more comprehensive training in dermatology within oncology programs, particularly in managing cancer-related skin toxicities [33]. Onco-dermatology, as a specialized interdisciplinary field, evolves as a niche discipline that bridges the gap between oncology and dermatology [16, 34]. The importance of having an onco-dermatologist on-site or in close collaboration with oncology teams has been emphasized in other studies, where the presence of such specialists significantly improved the timely management of skin toxicities, reducing interruptions in cancer treatment [25, 29, 35, 36]. Third, in addition to treatment-related challenges, oncologists face patient-centered obstacles in managing skin toxicities. Oncologists reported that patients may not view their skin issues as significant enough to mention during the visit because they are focused on cancer, which complicates proactive management, and that some patients perceive dermatologic treatments as purely esthetic, which reduces the likelihood of seeking appropriate care for skin toxicities. These findings align with previous research showing that patients may prioritize cancer treatment over dermatologic concerns [8] and that while skin problems are common, they are often given lower priority compared to clinical tumor responses or life-threatening side effects such as neutropenia [17, 37]. On the other hand, oncologists expressed concern regarding the psychological burden of skin toxicities like hair loss, which is well-known to cause significant emotional distress, affecting patients’ self-esteem and quality of life [38, 39]. This concern must, however, be related to anti-tumor treatments that can vary in skin toxicities. There are treatments that cause only mild skin toxicities, not painful, that do not impact the quality of life, while in other cases, there are toxicities that can be devastating to the patient's daily life, deteriorating their quality of life and self-esteem or representing the need for other treatments and medical visits, with the intake of additional drugs [17, 37]. In addition, the new anti-tumor therapies can represent long-term therapies, with cycles that can last a lifetime. In that case, even non-serious but long-lasting dermatological problems can modify the success of the treatment. As such, effective skin management is crucial for maintaining quality of life during cancer treatment. It’s important to remember that the management of skin toxicities in cancer patients varies greatly if it is entrusted to a dermatologist or an oncologist. Dermatologists use many drugs, including biological drugs, retinoids, many immunosuppressants that are not commonly used in oncology departments. This can significantly change the results in the management of adverse events, especially those induced by immunotherapy [40].

One of the key strengths of this study is that it is the first to address perspectives on how dermatologists and oncologists approach cancer-related skin toxicities in the Middle East. Additionally, this study used a mixed-methods design incorporating both quantitative and qualitative analysis, allowing for a more in-depth exploration of the challenges faced in clinical practice. However, there are limitations. The study did not gather demographic characteristics of participants, which could have provided more context for the findings. Furthermore, the generalizability of the results is limited by the small sample size and the potential for selection bias related to participant characteristics. In particular, dermatologists in this study represented general practitioners rather than specialized onco-dermatologists, and their experiences, depending on their exposure to oncology cases, the city where they practice, and the structure of their healthcare settings, may have influenced the reported frequency and comfort level in managing cancer-related skin manifestations. Despite this, all oncologists invited to participate responded, and the smaller sample size allowed for further qualitative analysis. Furthermore, it should be noted that not all anticancer drug toxicities were investigated; the study focused only on the most frequent ones. Nonetheless, the study provides valuable descriptive data that can guide future research and serve as a foundation for developing evidence-based strategies for managing skin toxicities in cancer patients in the Middle East.

## Conclusions

In conclusion, this study provides valuable insights into the differing perspectives, practices, and challenges of dermatologists and oncologists in managing skin manifestations in cancer patients. Our findings emphasize the need for increased collaboration between the two specialties, enhanced academic and clinical onco-dermatology education, and the integration of dermatologic expertise into cancer care settings. Future efforts should focus on developing standardized guidelines for the management of skin toxicities related to oncology treatments in a patient-centered care approach. Addressing the barriers to treatment, including the selection of dermo-cosmetic products, the severity of skin conditions, as well as the gaps in knowledge, could play a pivotal role in improving patient care.

## Supplementary Information

Below is the link to the electronic supplementary material.

ESM 1 (DOCX 20.1 KB)

## Data Availability

All data generated or analyzed during this study is included in this published article.

## Abbreviations

MEMC: Middle East Medical Convention
ASCO: American Society of Clinical Oncology
ESMO: European Society for Medical Oncology
EADV: European Academy of Dermatology and Venereology
