# Supplementary material for: Perspectives on managing skin manifestations in cancer patients: a multidisciplinary mixed-method survey of oncologists and dermatologists
Source: Support Care Cancer. 2026 Jan 11;34(2):82. doi: 10.1007/s00520-025-10254-w (PMC12790518; doi:10.1007/s00520-025-10254-w)
Supplement: Supplementary file 1 — (DOCX 20.1 KB) [file 520_2025_10254_MOESM1_ESM.docx]

**Perspectives on Managing Skin Manifestations in Cancer Patients:**

**A Multidisciplinary Mixed-Method Survey of Oncologists and Dermatologists**

This brief questionnaire aims to explore the experiences, practices, and challenges of healthcare professionals involved in managing skin manifestations among cancer patients. The survey is anonymous and will take approximately 5 minutes to complete. Your responses will contribute to improving collaboration between dermatology and oncology specialists in the region.
Please indicate your specialty below. You will then be directed to the corresponding set of questions.

**Please indicate your specialty:**

- Dermatologist
- Oncologist

**If Dermatologist:**

- **How often you consult in your clinic on a cancer patient with skin manifestations**:
  - Daily
  - Weekly
  - Monthly
  - Every quarter
  - Rarely
- **What are the most common complaints/manifestations you encounter:**
  - Dry skin
  - Nail changes
  - Photosensitivity
  - Skin discoloration
  - Hair loss
  - Scars
  - Hand of Foot syndrome
- **How comfortably would you treat their skin ailments:**
  - Very comfortable
  - Somehow comfortable
  - Not at all
- **In case you refrain from treating these patients, to which specialty you refer them:**
  - The treating oncologist
  - Another dermatologist
  - An onco-dermatologists
  - A GP
  - A pharmacist
- **What would keep you from treating those cases:**
  - Severity of the condition
  - Fear of inappropriate choice of treatments
  - Lack of knowledge/training in oncology patient care
  - Lack of knowledge in cancer friendly dermo-cosmetics products
  - Other

**If Oncologist:**

- **How frequently you encounter cancer patients developing skin pathologies:**
  - Daily
  - Weekly
  - Monthly
  - Every quarter
  - Rarely
- **What are the most common complaints/manifestations you encounter:**
  - Dry skin
  - Nail changes
  - Photosensitivity
  - Skin discoloration
  - Hair loss
  - Scars
  - Hand of Foot syndrome
- **How comfortably would you treat their skin ailments:**
  - Very comfortable
  - Somehow comfortable
  - Not at all
- **In case you refrain from treating these patients, to which specialty you refer them:**
  - Another dermatologist
  - An onco-dermatologists
  - A GP
  - A pharmacist
  - A nurse
- **What would keep you from treating those cases:**
  - Severity of the condition
  - Fear of inappropriate choice of dermo-cosmetics treatments
  - Lack of proper dermatology knowledge
  - Other
- **How frequent do you stop an anti-cancer treatment because of skin manifestations:**
  - 1 in 10 patients
  - 2 in 10 patients
  - Never
  - Other, Please specify: …………………………………………………………………………………….
- **Kindly list the top 3 challenges you face when treating skin manifestation of a cancer patient**:

………………………………………………………………………………………………………

………………………………………………………………………………………………………

………………………………………………………………………………………………………
